# Estrogen-dependent downregulation of hypoxia-inducible factor (HIF)-2α in invasive breast cancer cells

**DOI:** 10.18632/oncotarget.8866

**Published:** 2016-04-20

**Authors:** Jerry H. Fuady, Katrin Gutsche, Sara Santambrogio, Zsuzsanna Varga, David Hoogewijs, Roland H. Wenger

**Affiliations:** ^1^ Institute of Physiology and Zurich Centre for Human Physiology (ZIHP), University of Zurich, Zurich, Switzerland; ^2^ Institute of Surgical Pathology, University Hospital Zurich, Zurich, Switzerland; ^3^ Institute of Physiology, University of Duisburg-Essen, Essen, Germany

**Keywords:** ERα, HER2, histone deacetylation, hormone therapy, tumor oxygenation

## Abstract

The involvement of estrogen (E2) and hypoxia in tumor progression is well established. Hypoxia has been reported to activate and degrade estrogen receptor alpha (ERα) in breast cancer cells. Furthermore, E2 has been shown to regulate hypoxia-inducible factor (HIF)-1α protein, but its role in HIF-2α regulation remains largely unexplored. In this study, we found that both HIF-2α mRNA and protein were down-regulated in ER positive but not ER negative breast cancer cells upon treatment with E2. The analysis of 690 samples derived from 608 mixed and 82 triple-negative breast cancer patients revealed that high nuclear HIF-2α tumor levels are associated with a worse prognosis specifically in human epidermal growth factor receptor 2 (HER2) and hormone receptor positive patients. Consistently, ERα/HER2 positive breast cancer cells displayed less pronounced downregulation of HIF-2α by E2. Experiments using a histone deacetylase inhibitor indicate that the E2 mediated decrease in HIF-2α mRNA is due to transcriptional repression. A functional estrogen response element (ERE) was identified in the first intron of the gene encoding HIF-2α (*EPAS1*), suggesting transcriptional co-repressor recruitment by ERα. Our results demonstrate a novel modulation of HIF-2α in breast cancer cells, explaining the opposing regulation between HIF-1α and HIF-2α in hormone-responsive breast cancer.

## INTRODUCTION

The ability of cancer cells to adapt to micro-environmental tissue hypoxia is mainly mediated by hypoxia-inducible factors (HIFs), which affect every aspect of cancer progression, comprising metabolism, proliferation, inflammation, angiogenesis, metastasis and therapy resistance [1–4]. Transcriptionally active HIFs are heterodimers composed of a constitutively expressed β subunit and an oxygen labile HIF-1α or HIF-2α subunit, the stability and activity of which is regulated by prolyl-4-hydroxylase domain (PHD) and factor inhibiting HIF (FIH) enzymes [5]. Despite showing similar protein structures and having identical DNA recognition sequences, distinct - sometimes even opposite - functional roles of HIF-1α and HIF-2α in tumorigenesis have been reported [6–9]. We and others found different kinetics of hypoxic HIF-1α and HIF-2α protein induction and target gene expression [10, 11], further suggesting non-overlapping roles for these two related transcription factors.

Estrogens are steroid hormones and represent the primary female sex hormones, regulating diverse physiological processes in reproductive, mammary, cardiovascular, osseous, hepatic, and neuronal tissues [12–16]. The cellular effects of estrogens are mediated by two estrogen receptor (ER) isoforms, ERα and ERβ, which belong to the family of nuclear hormone receptors [17]. Ligand binding leads to the dissociation of heat shock proteins from the ER, which is followed by receptor dimerization and nuclear translocation. In the nucleus, the activated dimer complex binds to estrogen response elements (EREs) located within the regulatory regions of target genes [18].

Besides regulating numerous aspects of human physiology, estrogens also influence diverse pathophysiological processes, including the onset and progression of breast cancer [19]. Breast cancer is the most common cancer in women worldwide and the second most common cancer overall. Approx. 1.7 million new cases were diagnosed in 2012, which represents 12% and 25% of all new cancer cases and all cancers in women, respectively [20, 21]. The presence of elevated ERα levels in benign breast epithelium correlates with an increased risk of breast cancer, suggesting a role for ERα in breast cancer initiation [22]. 17β-estradiol (E2), the dominant circulating estrogen, regulates the growth of many breast tumors, and approx. 70% of breast cancers express ERα. Most of these ERα positive tumors depend on estrogen signaling for their growth and survival [23].

In conjunction with estrogen, hypoxia has been reported to play an important role in the development and progression of breast cancer [24–28]. In breast cancer cells, estrogen and hypoxia modulate the expression of genes involved in proliferation, differentiation, angiogenesis, metabolism and apoptosis [29–31]. Further studies revealed the presence of a cross-talk between estrogen signaling pathways and HIF-1α regulation in breast cancer [28, 32]. Estrogen has been reported to rapidly induce ERα-c-Src-PI3K interactions, which activates the PI3K/AKT/mTOR pathway and subsequent HIF-1α protein translation by phosphorylation of the p70 S6 kinase and 4EB-P1 [27]. Activation of G protein-coupled receptor GPR30 (GPER) by E2 triggers the GPER/EGFR/ERK/c-fos signaling pathway, leading to increased VEGF via HIF-1α upregulation [33]. Furthermore, ERα has been reported to directly induce HIF-1α transcription, which might modulate the anti-estrogen response in breast cancer treatment [32]. In contrast, ERβ has been reported to play an opposing role to ERα. Transcriptional activity of HIF-1 is inhibited by ERβ, which is mediated by ubiquitin-dependent degradation of HIF-β [27].

Despite the numerous studies on HIF-1α regulation by estrogen, the interaction between estrogen signaling and HIF-2α regulation is currently unknown. By immunohistochemical detection of HIF-2α in tissue microarrays of 282 invasive breast cancer cases we previously found that patients expressing high HIF-2α levels had a better overall survival rate compared to patients expressing low nuclear HIF-2α [11]. Our study was confirmed by the designation of HIF-2α in a list of genes associated with favorable outcome based on studies with different cohorts of breast cancer patients [34]. Here, we report a previously not recognized regulation of HIF-2α by estrogen, suggesting an inverse interplay between estrogen and HIF-2α signaling, which might be involved in breast cancer progression. These data provide a more complex picture of the role of HIF-2α in breast cancer, including receptor status and hormone dependent regulation.

## RESULTS

### E2 downregulates HIF-2α mRNA and protein levels in ERα positive but not ERα negative breast cancer cell lines

**Figure 1:**
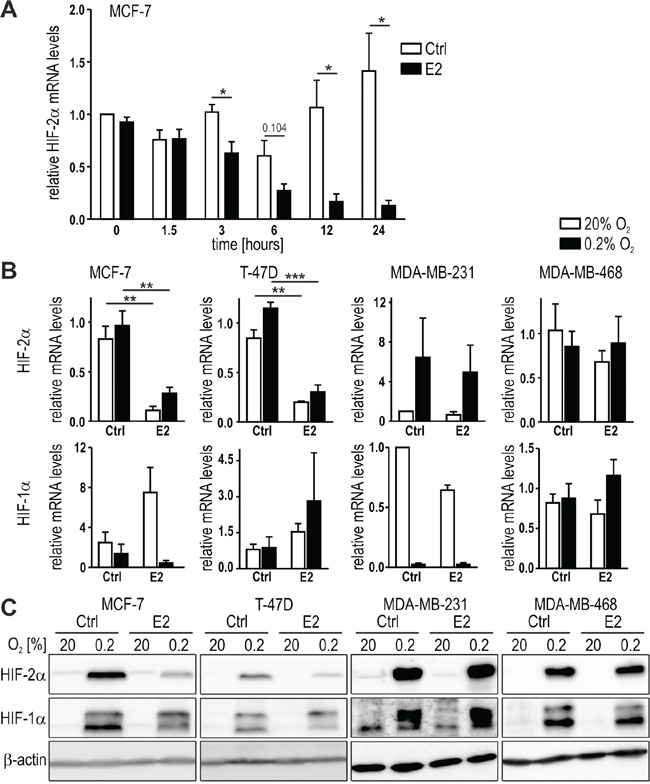
HIF-2α regulation by estrogen in breast cancer cell lines **A.** MCF-7 cells were treated with 10 nM E2 for the indicated time periods. HIF-2α mRNA levels were quantified by RT-qPCR, corrected for the endogenous L28 mRNA levels and normalized to the starting time point. **B.** ERα positive (MCF-7 and T-47D) and ERα negative (MDA-MB-231 and MDA-MB-468) breast cancer cell lines were treated with 10 nM E2 for 24 hours under normoxic or hypoxic conditions. HIF-2α mRNA levels were quantified as above. Shown are mean values ± standard errors of the mean (SEM) of three to four independent experiments. For statistical evaluation, the effects of E2 treatment were compared with the ethanol solvent control (Ctrl) treatment. *P<0.05; **P<0.01; ***P<0.001. **C.** HIF-2α protein was detected by immunoblotting and constitutively expressed β-actin was used as loading and blotting control.

We previously reported a HIF-2 specific regulation of WISP-2 expression in breast cancer cells [35]. Because WISP-2 is a known ERα target gene [36], we aimed for the analysis of the cooperation between estrogen and oxygen signaling. Therefore, the time-dependent effect of E2 treatment on HIF signaling in MCF-7 cells was investigated. Unexpectedly, HIF-2α mRNA levels were progressively reduced with increasing time of E2 treatment (Figure 1A). Maximal inhibitory effects were reached after 12 to 24 hours, and the latter time-point was selected for all subsequent experiments, also based on our previous observation that HIF-2α protein levels in MCF-7 cells are expressed maximally after 24 hours of hypoxic exposure [11].

We further tested the effects of E2 on HIF-2α in additional breast cancer cell lines, including another luminal-like ERα positive (T-47D), a basal B-like ERα negative (MDA-MB-231), and a basal A-like ERα negative (MDA-MB-468) cell line. Both, HIF-2α mRNA (Figure 1B) and hypoxically stabilized protein (Figure 1C) levels were downregulated in the ERα positive MCF-7 and T-47D but not in the ERα negative MDA-MB-231 and MDA-MB-468 cell lines. Exposure to hypoxia (0.2% O_2_) for 24 hours generally did not affect the HIF-2α mRNA levels, except in MDA-MB-231 cells where HIF-2α mRNA was induced. However, E2 did not alter this cell type-specific HIF-2α mRNA regulation. Neither HIF-1α mRNA (Figure 1B) nor hypoxically stabilized HIF-1α protein levels (Figure 1C) were significantly affected by 24 hours E2 treatment. These data suggest that HIF-2α is specifically downregulated by E2-ERα signaling on the mRNA level, which resulted in corresponding changes on the protein levels.

### The selective estrogen receptor modulator tamoxifen prevents ERα-dependent HIF-2α downregulation

**Figure 2:**
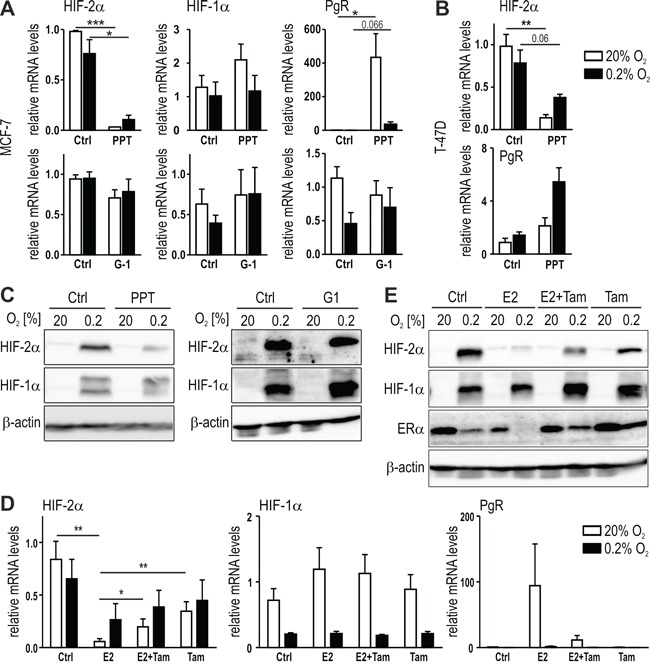
ERα-mediated regulation of HIF-2α by estrogen MCF-7 (**A** and **C**) or T-47D **(B)** cells were treated for 24 hours with 10 nM PPT, an ERα-specific agonist with approx. 400 times higher affinity towards ERα than towards ERβ, or 1 μM G1 (MCF-7 only), a GPER-specific agonist, under normoxic or hypoxic conditions. The mRNA (**A** and **B**) and protein (**C**) levels were determined by RT-qPCR and immunoblotting, respectively. **D** and **E.** Tamoxifen (5 μM) was added to MCF-7 cells with or without 10 nM E2 and mRNA (**D**) and protein (**E**) levels were determined. Shown are mean mRNA values ± SEM of three independent experiments. For statistical evaluation, the effects of E2 treatment were compared with DMSO solvent control (Ctrl) treatment. *P<0.05; ***P<0.001.

To further investigate the involvement of estrogen signaling in HIF-2α and HIF-1α regulation, MCF-7 cells were treated for 24 hours with the ERα-specific agonist propyl pyrazole triol (PPT) and with a selective agonist for GPR30 (G-1) under normoxic or hypoxic conditions. HIF-2α but not HIF-1α mRNA levels were downregulated by PPT but not by G-1 (Figure 2A). Progesterone receptor (PgR) was included as a positive control for ERα activation by PPT. Hypoxia inhibited PgR induction in MCF-7 cells, probably due to the known hypoxic degradation of ERα protein in these cells [37], as also shown below in Figure 2E. Downregulation of HIF-2α mRNA by PPT was confirmed in T-47D cells (Figure 2B). Similar data were obtained in MCF-7 cells on the protein level (Figure 2C), suggesting the involvement of ERα but not GPR30 in estrogen-mediated HIF-2α inhibition.

The current first line therapy of ER positive breast cancer patients is the treatment with the selective estrogen receptor modulator (SERM) tamoxifen. Therefore, MCF-7 cells were treated with E2, with or without tamoxifen, under normoxic or hypoxic conditions. As shown in Figure 2D, tamoxifen at least partially prevented HIF-2α mRNA downregulation by E2, while HIF-1α mRNA levels were neither affected by E2 nor tamoxifen. *Vice versa*, PgR mRNA upregulation by E2 was partially prevented by tamoxifen. Corroborating the findings on the mRNA levels, tamoxifen also partially prevented the E2-mediated downregulation of the hypoxically stabilized HIF-2α protein levels (Figure 2E). The slight HIF-2α but not HIF-1α protein downregulation by tamoxifen may be explained by the similarly decreased mRNA levels. However, these findings suggest that SERMs can reverse the inhibitory effects of estrogen on HIF-2α.

### Inverse correlation between HIF-2α and ERα mRNA levels in breast cancer

**Figure 3:**
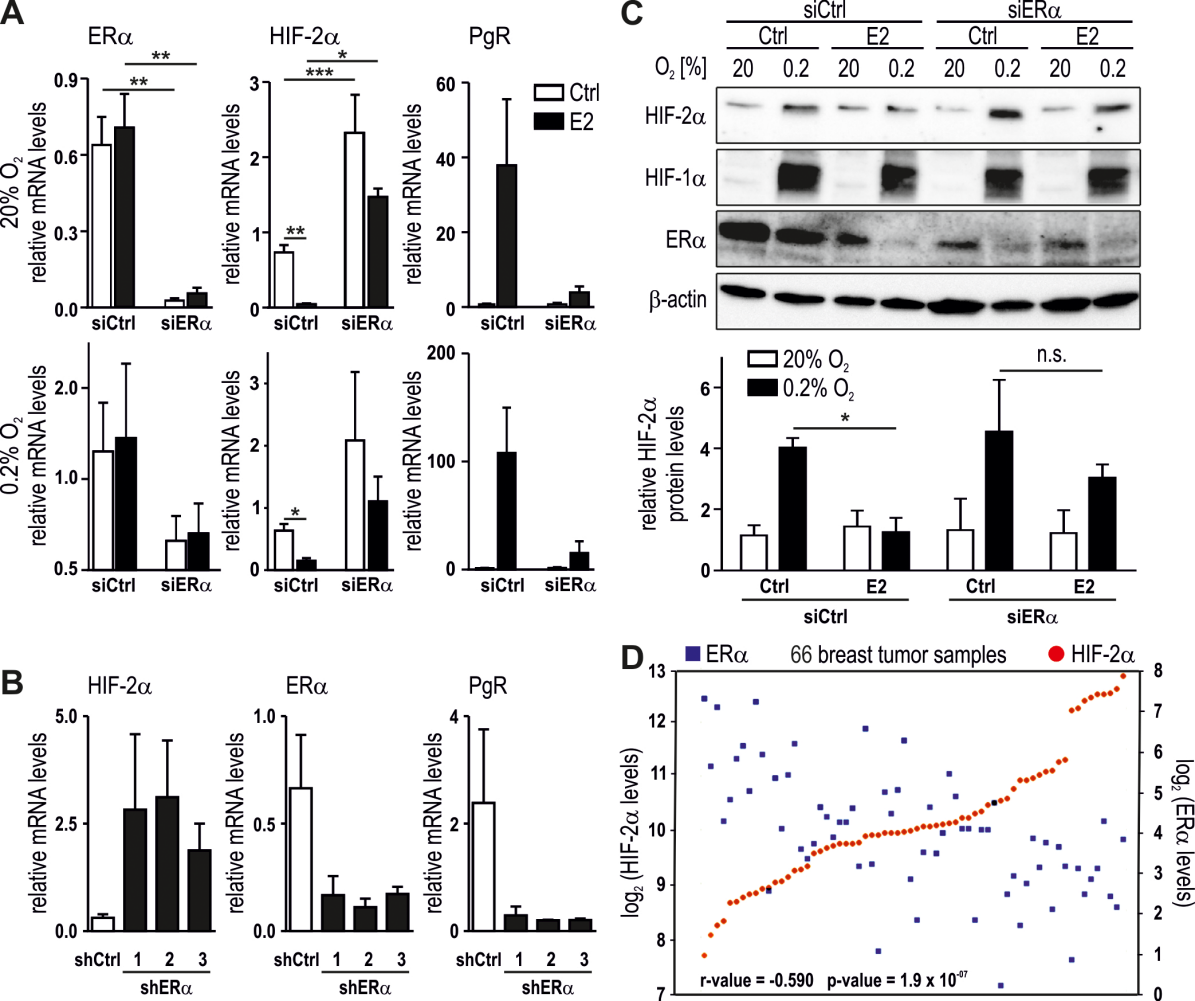
Reciprocal regulation of HIF-2α and ERα ERα was knocked down in MCF-7 cells using siERα **(A** and **C)** or shERα **(B)** and then treated with 10 nM E2 under normoxic or hypoxic conditions. mRNA and protein levels were subsequently determined by RT-qPCR (**A** and **B**) and immunoblotting (**C**, upper panel), respectively. Shown are mean mRNA (**A** and **B**) and protein (**C**, lower panel) values ± SEM of three independent experiments. For statistical evaluation, the effects of ERα silencing were compared with siCtrl cells; the effects of E2 treatment were compared with ethanol solvent control (Ctrl) treatment. n.s., not significant; *P<0.05; **P<0.01. **D.** Microarray data from public databases were compiled using the R2 genomic analysis tool. Significance of the negative correlation between HIF-2α (red dots) and ERα (blue squares) mRNA levels was assessed by one-way ANOVA.

To complement the pharmacological E2 receptor modulation shown above, ERα was downregulated by siRNA treatment of MCF-7 cells. Knockdown efficiency was confirmed by a 96% decrease of the constitutive ERα mRNA levels as well as by a 97% decrease of the E2-induced mRNA levels of the ERα target gene PgR (Figure 3A). Both, basal and E2-inhibited HIF-2α mRNA levels were significantly increased in siERα but not siCtrl treated MCF-7 cells. While E2 strongly downregulated HIF-2α mRNA levels in siCtrl cells, the remaining slight downregulation in siERα cells was not significant (Figure 3A). Similar results were obtained in MCF-7 cells stably transfected with three independent shRNA constructs which resulted in 74-83% ERα knockdown efficiency and in a strong decrease of PgR, whereas constitutive HIF-2α mRNA levels were enhanced (Figure 3B). Probably due to the remaining ERα, blockade of the E2-mediated HIF-2α downregulation and PgR upregulation was rather inefficient in shERα compared with siERα cells (data not shown). These findings were corroborated on the protein level where hypoxically stabilized HIF-2α but not HIF-1α is clearly less downregulated by E2 in siERα than in siCtrl treated MCF-7 cells (Figure 3C).

Because the results shown above suggest an inverse correlation between HIF-2α and ERα mRNA levels in breast cancer cell lines, we explored transcriptome data from various clinical breast cancer studies employing the R2 microarray analysis and visualization platform (http://hgserver1.amc.nl/cgi-bin/r2/main.cgi). A study of 66 tamoxifen-treated breast cancer patients showed a significant negative correlation between the expression of HIF-2α and ERα (Figure 3D). Several additional datasets from three different microarray studies including 136, 116 and 61 breast cancer patients, respectively, displayed a similar inverse correlation (Supplementary Figure 1).

### High HIF-2α expression is a negative prognostic factor in HER2 positive breast cancer

**Figure 4:**
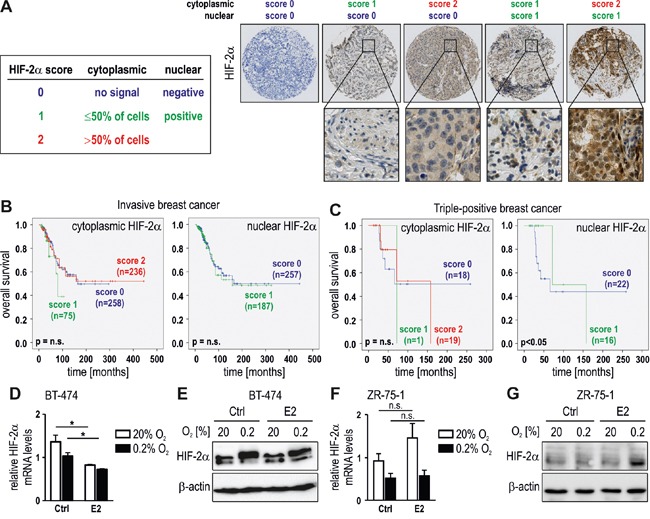
HIF-2α as a prognostic factor in HER2 positive breast cancer **A.** Scoring system and representative images for cytoplasmic and nuclear HIF-2α staining of invasive breast cancer tissues. **B.** HIF-2α was scored in tissue microarrays of 608 invasive breast cancer cases (excluding triple-negative cases) and displayed in Kaplan-Meier survival curves. **C.** Survival curves of hormone receptor positive and HER2 overexpressing breast cancer patients according to HIF-2α scores. **D** to **G.** Triple-positive BT-474 and ZR-75-1 breast cancer cells were treated with 10 nM E2 for 24 hours under normoxic or hypoxic conditions, and mRNA **(D, F)** and protein **(E, G)** levels of HIF-2α were determined. Shown are mean mRNA values ± SEM of three independent experiments. For statistical evaluation, the effects of E2 treatment were compared with ethanol solvent control (Ctrl) treatment. *P<0.05; n.s., not significant.

To study the role of HIF-2α in breast tumorigenesis, two tissue microarrays containing invasive breast cancer tumor samples derived from 690 breast cancer patients with primary breast cancer were immunostained for HIF-2α. The tissue microarrays contained areas from invasive breast cancer belonging to ERα positive, HER2 positive or triple-negative cases, as described previously [38]. The signals from the invasive breast cancer samples in the tissue microarrays were scored based on the presence or absence of cytoplasmic and nuclear HIF-2α staining as exemplified in Figure 4A. When all 690 cancer cases were included, neither cytoplasmic nor nuclear HIF-2α correlated with overall survival (Figure 4B). Stratification of cytoplasmic and nuclear HIF-2α to nodal status, tumor stage and histological grade showed no correlation with overall survival (Supplementary Figure 2A-2F). Also the analysis of the 82 triple-negative breast tumor samples did not reveal any significant association between HIF-2α levels and overall survival (Supplementary Figure 2G). However, stratification of nuclear HIF-2α according to the HER2 positivity status in 38 hormone receptor positive breast cancer patients displayed a significant negative correlation with overall survival in patients with high nuclear HIF-2α (Figure 4C).

Because the ERα positive breast cancer cell lines used in Figure 1 are not overexpressing HER2, we analyzed the hormone receptor and HER2 positive breast cancer cell line BT-474. While treatment with 10 nM E2 for 24 hours downregulated HIF-2α mRNA levels (Figure 4D), the fold inhibition was less pronounced than in MCF-7 and T-47D cells (Figure 1), and cannot be detected on the protein level (Figure 4E). Whereas strongly HER2 overexpressing cell lines are quite rare, ZR-75-1 has been reported to be HER2 positive [39]. We hence repeated these experiments in ZR-75-1 cells which showed no HIF-2α downregulation by E2, neither on the mRNA (Figure 4F) nor on the protein (Figure 4G) level, confirming the results obtained in BT-474.

In conclusion, diminished downregulation of HIF-2α by E2 in HER2 high ERα positive BT-474 and ZR-75-1 cells is consistent with the correlation between high HIF-2α levels and worse survival of hormone receptor and HER2 positive breast cancer patients.

### ERα-dependent HIF-2α downregulation is independent of mutual HIFα inhibition or HIF-2α mRNA stability

**Figure 5:**
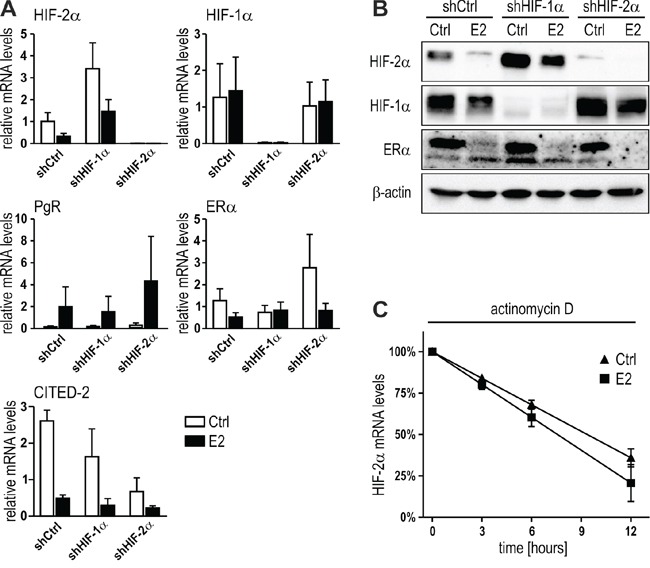
Mutual HIF-α inhibition or mRNA stability are not involved in E2-dependent HIF-2α regulation **A.** MCF-7 shCtrl, shHIF-1α and shHIF-2α cells were treated with 10 nM E2 for 24 hours under hypoxic conditions and mRNA levels determined by RT-qPCR. Shown are mean values ± SEM of three independent experiments. The effects of E2 treatment were compared with ethanol solvent control (Ctrl) treatment. **B.** Immunoblotting of MCF-7 cells treated with E2 as above. **C.** MCF-7 cells were pre-treated with 1 μM actinomycin D for 1 hour before adding 1 nM E2. HIF-2α mRNA was quantified by RT-qPCR and normalized to the initial levels (100%). No significant difference between the linear regression slopes of the DMSO solvent control (Ctrl) and E2 treatment was found (n = 3).

To address the functional mechanism underlying HIF-2α inhibition by E2/ERα, we investigated known candidate HIF-2α inhibitors. We and others previously reported the mutual inhibition of HIF-1α and HIF-2α [40, 7]. Because E2 has been shown to induce HIF-1α expression [33], we hypothesized that increased HIF-1α might subsequently decrease HIF-2α. This hypothesis was tested by adding E2 to stably HIF-1α or HIF-2α depleted MCF-7 cells. As expected, shHIF-1α MCF-7 cells express higher levels of HIF-2α mRNA, whereas shHIF-2α had no effect on HIF-1α mRNA levels in this cell line (Figure 5A). Both, PgR and ERα remained largely unaffected by either HIF-1α or HIF-2α knockdown, and E2 downregulated HIF-2α in shHIF-1α to the same extent as in shCtrl cells. Also CITED-2, a preferential HIF-2 target gene [41, 10], was still downregulated by E2 in shHIF-1α MCF-7 cells (Figure 5A). Similar results were obtained on the protein level (Figure 5B), suggesting that mutual HIFα inhibition does not play any role in E2/ERα-dependent HIF-2α downregulation.

Because E2/ERα-mediated HIF-2α mRNA downregulation could be due to either transcriptional repression or decreased mRNA stability, we next analyzed the decrease of HIF-2α mRNA following RNA polymerase II inhibition by actinomycin D. As shown in Figure 5C, HIF-2α mRNA decay was not significantly different in the presence of E2 compared with solvent control, suggesting that HIF-2α mRNA levels were transcriptionally decreased by E2.

### Histone deacetylation is involved in HIF-2α inhibition by E2

**Figure 6:**
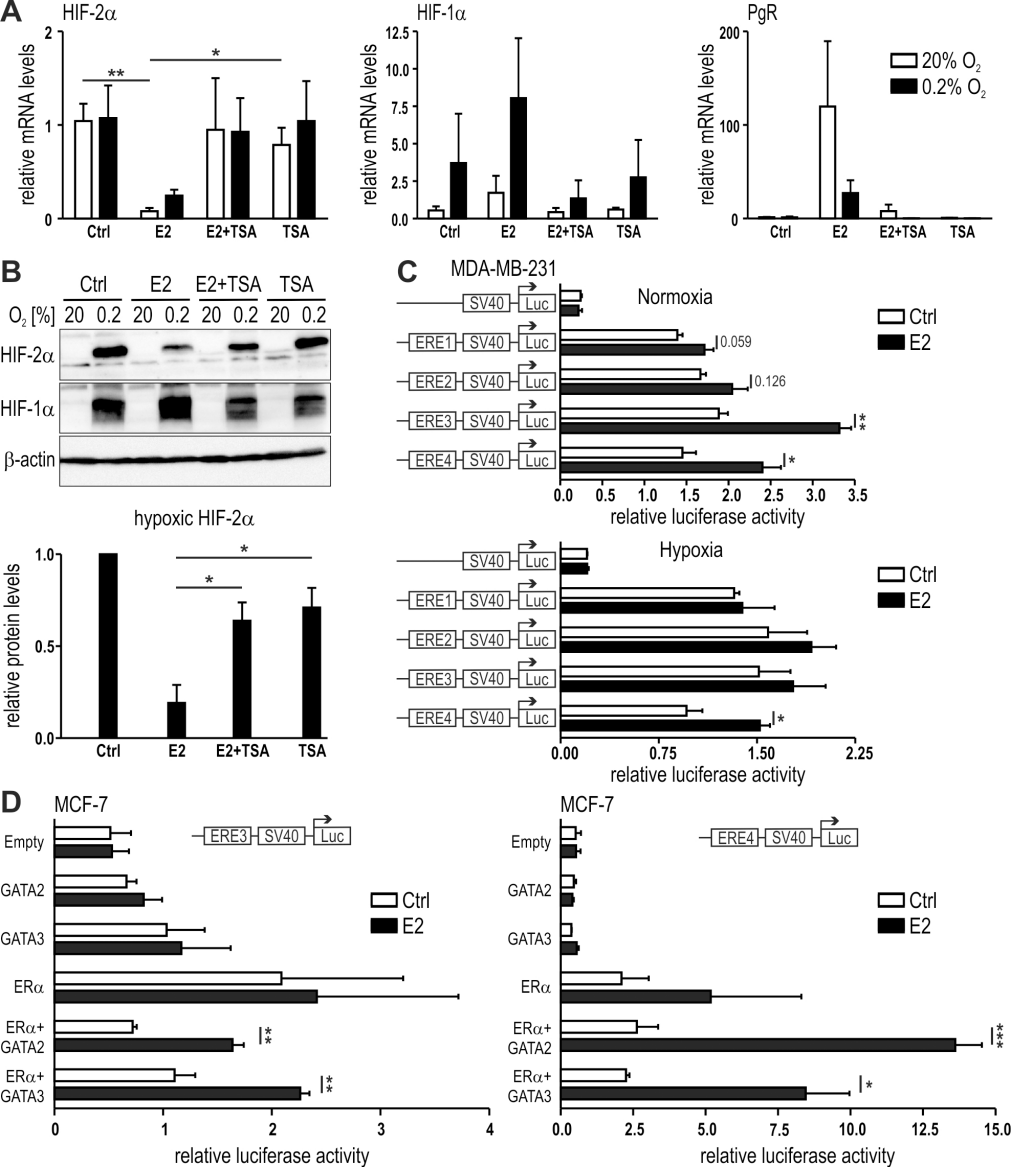
Role of histone deacetylation in E2-dependent HIF-2α regulation MCF-7 cells were treated with 10 nM E2 with or without 250 nM trichostatin A (TSA), an inhibitor of HDAC. mRNA **(A)** and protein **(B)** levels were determined by RT-qPCR and immunoblotting, respectively. **C.** Dual luciferase reporter gene assays were performed with MDA-MB-231 cells transiently co-transfected with a constitutively expressed *Renilla* luciferase plasmid, an *EPAS1*-derived ERE1 to 4 driven firefly reporter gene plasmid, and an ERα overexpression vector. **D.** Luciferase reporter gene assays were performed with MCF-7 cells transiently co-transfected with a constitutively expressed *Renilla* luciferase plasmid, an *EPAS1*-derived ERE3 or ERE4 driven firefly luciferase reporter gene plasmids, and expression vectors for ERα, GATA-2 or GATA-3. Shown are mean values ± SEM of three independent experiments. For statistical mRNA evaluation **(A)**, the effects of E2 alone were compared with DMSO solvent control (Ctrl) or with TSA or with TSA+E2 treatment. For statistical protein evaluation (**B**, lower panel), the values were normalized to the DMSO solvent control (Ctrl) and the effects of E2 alone were compared with TSA or with TSA+E2 treatment. For dual-luciferase reporter gene assays (**C** and **D**), three independent experiments were performed in triplicates. The relative luciferase activity was obtained by dividing the firefly luciferase values by the corresponding *Renilla* luciferase values. For statistical evaluation, the effects of E2 treatment were compared with ethanol solvent control (Ctrl) treatment. *P<0.05; **P<0.01; ***P<0.001.

Histone deacetylation is one of the prerequisites for chromatin remodeling and regulation of gene expression. In order to study the role of histone deacetylation in HIF-2α transcriptional regulation, MCF-7 cells were treated with E2 in the absence or presence of the class I and II mammalian histone deacetylase (HDAC) inhibitor trichostatin A (TSA). While TSA alone had no effect, it prevented HIF-2α mRNA (Figure 6A) and protein (Figure 6B) downregulation by E2, suggesting that HDACs are involved in HIF-2α regulation by E2.

According to published chromatin immunoprecipitation-sequencing (ChIP-seq) data [17], ERα binds to four distinct regions within the first intron of the gene encoding HIF-2α (*EPAS1)* upon E2 treatment (Supplementary Figure 3A). *In silico* analysis of the ChIP-seq information deposited in the UCSC-integrated ENCODE database revealed that these regions contained conserved estrogen response element (ERE) binding motifs. As shown in Supplementary Figure 3B, all four regions displayed robust DNaseI hypersensitivity (reflecting open chromatin) and the methylated and acetylated histone marks H3K4Me1 and H3K27Ac, respectively (reflecting active enhancers) but not H3K4Me3 (reflecting active promoters). HDAC binding may be transient and was only found in ERE2 (HDAC2). Specifically, in K562 erythroleukemia cells HDAC1/2 binding to ERE3 and HDAC1 binding to ERE4 but no HDAC binding to ERE1 and ERE2 could be detected (Supplementary Figure 3B). In T-47D breast cancer cells, ERE4 also displayed binding of ERα (data not shown). Interestingly, transcription factor (TF) ChIP-seq data further revealed the binding of GATA-2 and GATA-3, established transcriptional repressors [42–45], at ERE3 and ERE4 (Supplementary Figure 3B). Moreover, ERα also binds to an ERE within the *HIF1A* gene, overlapping with GATA-3 binding (data not shown).

To independently analyze the binding of ERα to the EREs 1 to 4 of the *EPAS1* gene, we evaluated the potential of the ERE DNA fragments (as indicated by red bars in Supplementary Figure 3A) to regulate firefly luciferase reporter gene expression driven by the heterologous SV40 promoter. The reporter gene constructs were transiently transfected into MDA-MB-231 cells together with an ERα overexpression vector. Transfected cells were treated with E2 for 24 hours under normoxic or hypoxic conditions and the luciferase activities were determined. ERE1 and ERE2 had no effects but ERE3 and ERE4 significantly enhanced E2-induced reporter gene activity in normoxia and ERE4 in hypoxia (Figure 6C). Whereas this result using “non-chromatinised” bacterial DNA is opposing to the endogenous HIF-2α mRNA regulation by E2, it still provides further evidence for functional interaction between activated ERα and distinct EREs of the *EPAS1* gene.

Plasmids containing ERE3 and ERE4 were then transfected into another breast cancer cell line (MCF-7), with or without ERα, GATA-2 or GATA-3. While co-transfection of the reporter genes together with ERα or GATA overexpression vector alone did not result in significant induction of luciferase activities upon E2 treatment, co-overexpression of ERα together with GATA-2 or GATA-3 resulted in significant E2-dependent activation of luciferase activity in normoxia and hypoxia (Figure 6D). Taken together, these results indicate that E2-activated ERα locates to at least one ERE within the *EPAS1* gene and recruits several transcriptional co-factors, including GATA factors and HDACs, leading to transcriptional repression of the *EPAS1* gene.

## DISCUSSION

Cross-talk between estrogen signaling and hypoxia-dependent signaling pathways has previously been reported, focussing on the interactions between estrogen signaling and HIF-1α regulation [27, 32, 37, 46, 47]. In the present study, we report for the first time the association between estrogen signaling and HIF-2α regulation. Estrogen signaling is an essential component of breast cancer progression as indicated by the prevalence of ERα overexpression in breast cancer patients [48]. Hypoxia represents another major factor in breast cancer progression, and the interaction between these two signaling pathways hence is of major clinical importance [4].

In this study, we observed an ERα-dependent downregulation of HIF-2α mRNA and protein levels by E2. Cell lines with different ERα status, pharmacological and RNA interference experiments confirmed the requirement of ERα for the E2 effects on HIF-2α. Higher constitutive expression of HIF-2α both on the mRNA and protein levels in ERα depleted MCF-7 was phenocopied in microarray data of breast cancer patients with different ERα levels. This observation suggests a constitutive ERα-dependent suppression of HIF-2α expression, which is strengthened by hormonal ERα activation. Of note, the E2-induced HIF-2α repression was almost completely abrogated in hormone receptor and HER2 triple-positive cells. While it is currently unclear how HER2 interferes with HIF-2α regulation, HER2 signalling is known to induce HIF-1α by PI3K/Akt/mTOR signalling [49–51], and a similar mechanism might also overcome E2-mediated HIF-2α repression.

The ERα utilizes multiple mechanisms to either induce or suppress transcription of its target genes, which include direct binding of ligand-activated receptor to the DNA at the EREs, followed by recruitment of transcriptional co-regulators [52, 53]. Also an indirect modulation via sequestration of general transcriptional components has been suggested [54]. ERα activation is usually assumed to be associated with increased gene expression, however in fact almost 70% of the genes regulated by E2 are down-regulated in MCF-7 [55]. Our observation of HIF-2α down-regulation by E2 is thus consistent with the majority of genes being downregulated upon E2 treatment of MCF-7 cells.

Currently, we have no definitive explanation for the mechanism by which ERα inhibits HIF-2α. The state of histone acetylation is a predictor of gene activity, and HDACs are known to repress gene expression by modulating the conformational state of the chromatin. Furthermore, HDACs have been reported to be involved in tumorigenesis [56, 57]. Estrogen-mediated repression of the cell cycle inhibitor Reprimo (RPRM) required the interactions between ERα, HDAC7 and FoxA1 [58]. In addition, expression of the gene encoding human uridine diphosphate glucuronosyltransferase (*UGT1A*) was inhibited in the presence of ERα. ChIP assays further demonstrated the recruitment of ERα, HDAC1 and HDAC2 to the xenobiotic response elements of *UGT1A* promoters during gene repression [59]. Moreover, tamoxifen-bound ERα has been reported to recruit HDAC to silence gene transcription of ERα targets [60]. Interestingly, tamoxifen treatment of MCF-7 cells has recently been shown to significantly increase HDAC1 binding on the ERE of HIF-1α [32]. In our study, treatment of MCF-7 cells with E2 and TSA abolished ERα-dependent HIF-2α regulation. We identified several EREs within the regulatory region of the *EPAS1* gene, and ERE4 interacts with ERα in both MCF-7 and T-47D cells. ERE4 can also interact with GATA-2 and GATA-3, consistent with recent studies revealing substantial enrichment of GATA3 binding to ERα occupied DNA regions [61, 62]. Furthermore, besides inducing gene expression, GATAs have been reported to also exert repressive functions [42–45]. Luciferase reporter gene assays demonstrated a functional interaction between GATA-2/3 and the *EPAS1* ERE4, suggesting a role of ERE4 in ERα-dependent HIF-2α regulation.

In conclusion, hormone activation may lead to enhanced ERα dimerization, binding to *EPAS1* ERE4, recruitment of transcriptional co-factors, including GATAs and HDACs, and repression of gene expression. The lack of ERα in triple-negative breast cancer cells allows for constitutively higher HIF-2α and prevents estrogen-mediated HIF-2α downregulation seen in ERα positive breast cancer cells. This model is consistent with the inverse correlation between ERα and HIF-2α mRNA levels, which we observed in several breast cancer gene array studies.

Intriguingly, the current study using 690 breast cancer tissue samples could only partially confirm the previously reported positive correlation between HIF-2α protein levels and overall patient survival [11]. In the previous study, we analyzed a smaller cohort of 282 invasive breast cancer cases containing mainly ERα positive and HER2 negative luminal A/B samples, with only 13 cases being HER2 positive [11]. In the current study, we used a different and much larger cohort, including 90 HER2 positive cases and 82 triple-negative samples. Moreover, different antibodies, pretreatment protocols and the nuclear vs. cytoplasmic signal scoring scheme applied in the current study may have contributed to the incomplete overlap between the results of the two studies. The correlation between high nuclear HIF-2α and shortened overall survival in the HER2 and hormone receptor positive patient sub-cohort may be due to the attenuated downregulation of HIF-2α upon E2 treatment in HER2 positive breast cancer cells.

## MATERIALS AND METHODS

### Reagents

E2 (17β-estradiol), PPT (propyl pyrazole triol), tamoxifen, fulvestrant and actinomycin D were purchased from Sigma-Aldrich (St Louis, MO, USA). G-1 was purchased from Tocris Biosciene (Bristol, UK). Reagents were dissolved in ethanol or dimethyl sulfoxide (DMSO). Antibodies against the following proteins were used: HIF-1α (BD Transduction Laboratories, Allschwil, Switzerland), HIF-2α (immunoblotting: Abnova Corporation, Taiwan; immunohistochemistry: Abcam, Cambridge, UK), ERα (Santa Cruz Biotechnology, Dallas, TX, USA), and β-actin (Sigma-Aldrich).

### Cell culture and treatments

The human breast cancer cell lines MCF-7, T-47D, MDA-MB-231, MDA-MB-468, ZR-75-1 and BT-474 were cultured in high-glucose Dulbecco's Modified Eagle's Medium (Sigma-Aldrich). Before experiments, the cells were maintained in phenol red-free DMEM supplemented with 10% charcoal-treated FCS (Gibco, Thermo Fischer Scientific, Waltham, MA, USA) for 1 to 2 days. Cells were treated with E2 or vehicle control (0.1% ethanol or 0.1% DMSO) alone or in combination with other ligands for 24 hours under normoxic and hypoxic conditions. Hypoxic experiments were performed at 0.2% oxygen and 5% CO_2_ in a gas-controlled glove box (Invivo2 400, Baker Ruskinn, Bridgend, UK) as described previously [63].

### mRNA and protein detection and quantification

Total cellular RNA was extracted as previously described [11]. Total RNA (2 μg) was reverse transcribed (RT) using AffinityScript reverse transcriptase (Agilent, Santa Clara, CA, USA) and complementary DNA (cDNA) levels were estimated by quantitative polymerase chain reaction (qPCR) using the primers listed in Supplementary Table 1A and a SYBR^®^ Green qPCR reagent kit (Sigma-Aldrich) in a MX3000P light cycler (Agilent). Transcript levels were calculated by comparison with a calibrated standard and expressed as ratios relative to ribosomal protein L28 mRNA levels. Immunoblotting, signal imaging and quantification were performed as previously reported [64]. Values were normalized to a β-actin loading control. Breast cancer tissue microarray analysis was performed as previously described [38].

### Plasmid construction and reporter gene assays

DNA fragments containing the *EPAS1*-derived EREs were generated by PCR using the primers listed in Supplementary Table 1B, and cloned into the pGL3-promoter luciferase reporter vector (Promega Corporation, Madison, WI, USA). A cDNA encoding human ERα cloned into the pCMV5 mammalian expression vector was kindly provided by A. Odermatt (Basel, Switzerland). Human GATA-2, GATA-3, and GATA-4 cloned into pcDNA3.1 were kindly provided by C. Dame (Berlin, Germany). Dual luciferase reporter gene assays were performed as described previously [64].

### *In silico* expression data

The R2 database (http://hgserver1.amc.nl/cgi-bin/r2/main.cgi) was searched using the across datasets and 2D gene overview options for correlations between *EPAS1* and *ESR1* in Affymetrix HG-U133plus2.0 based expression profiles normalized using MAS5. The following GEO IDs were employed: GSE29431 (r-value=−0.590, p-value=1.9e-07, 66 samples), GSE28844 (r-value=−0.349 p-value=5.8e-03, 61 samples), GSE12093 (r-value=−0.438 p-value=9.9e-08, 136 samples) and GSE5462 (r-value=−0.313 p-value=6.2e-04, 116 samples).

### Statistical analysis

If not indicated otherwise, unpaired Student's *t-tests* were applied. Differences between two values at the *P*<0.05 level were considered to be statistically significant.

## SUPPLEMENTARY FIGURES AND TABLES


