# Correction: Estrogen-dependent downregulation of hypoxia-inducible factor (HIF)-2α in invasive breast cancer cells

**DOI:** 10.18632/oncotarget.16388

**Published:** 2017-03-20

**Authors:** Jerry H. Fuady, Katrin Gutsche, Sara Santambrogio, Zsuzsanna Varga, David Hoogewijs, Roland H. Wenger

**Present:** The wrong symbol font was used in preparation of the PDF version of this manuscript.

**Correct:** The PDF was replaced with the correct version. Please see the original manuscript for download link.

Original article: Oncotarget. 2016; 7:31153-65. doi: 10.18632/oncotarget.8866.

