# Supplementary material for: Estrogen-dependent downregulation of hypoxia-inducible factor (HIF)-2α in invasive breast cancer cells
Source: Oncotarget. 2016 Apr 20;7(21):31153–65. doi: 10.18632/oncotarget.8866 (PMC5058746; doi:10.18632/oncotarget.8866)
Supplement: Supplementary file 1 [file oncotarget-07-31153-s001.pdf]

SUPPLEMENTARY FIGURES AND TABLE

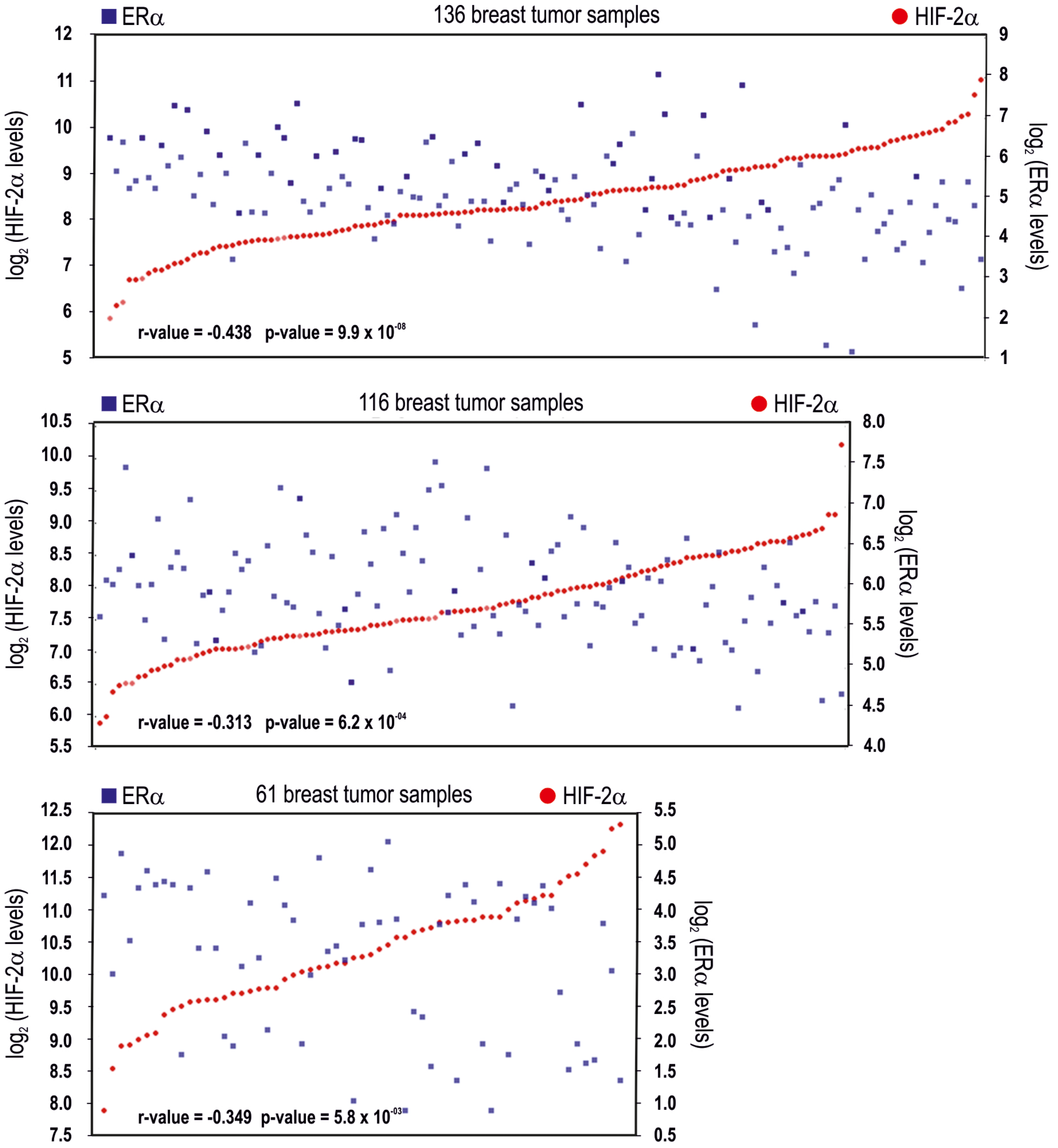

Supplementary Figure S1: Negative association between ERα and HIF-2α in breast cancer. Microarray data from three independent studies were compiled using the R2 genomic analysis tool. HIF-2α mRNA levels negatively correlate with ERα levels in breast cancers in all studies analysed, as assessed by one-way ANOVA.

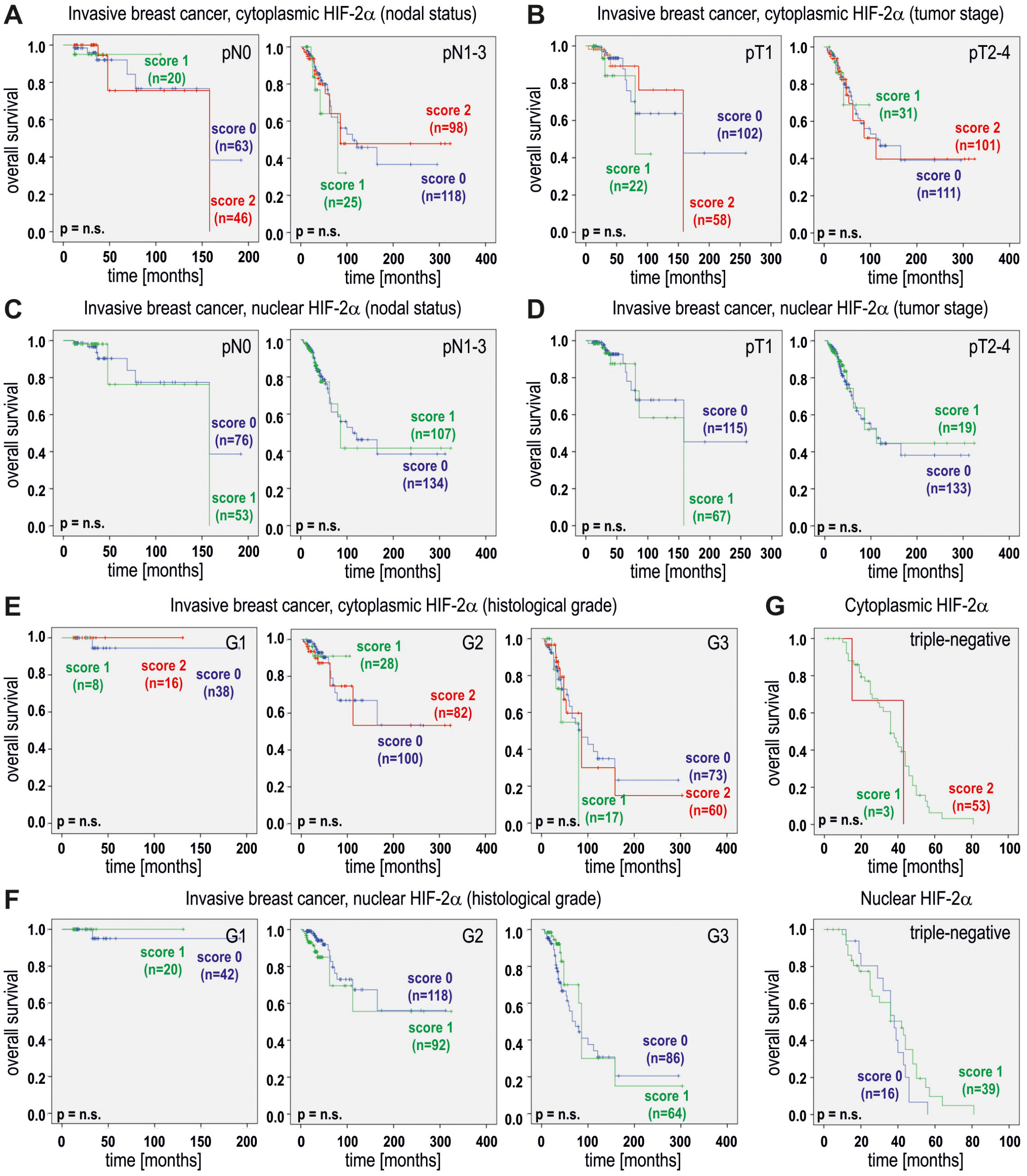

Supplementary Figure S2: Kaplan-Meier survival curves related to HIF-2α scores. Kaplan-Meier survival curves of cytoplasmic (**A**, **B**, **E** and **G**) and nuclear (**C**, **D**, **F** and **G**) HIF-2α scores in breast cancer samples stratified according to nodal status (**A** and **C**), tumor stage (**B** and **D**), histological grade (**E** and **F**), or in triple-negative breast cancer (**G**).

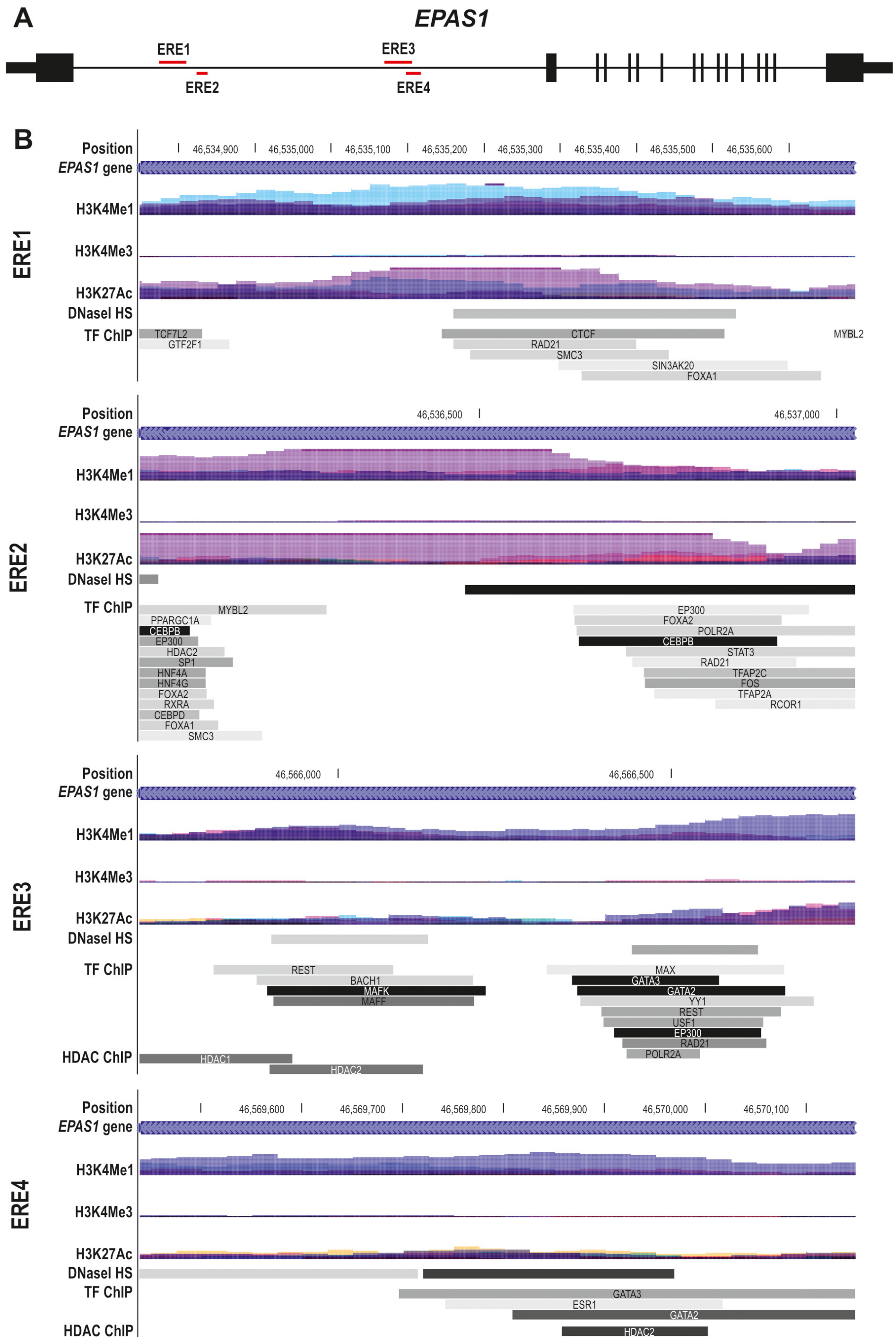

Supplementary Figure S3: Potential ERE sites in the *EPAS1* gene. A. Schematic representation of the *EPAS1* gene, indicating four estrogen response elements (EREs) located within the first intron. **B.** UCSC-integrated ENCODE data of the four ERE-containing loci (ERE1-4) of the *EPAS1* gene. In the transcription factor (TF) ChIP-sequencing track, gray boxes represent conventional ENCODE codes and encompass the peaks of TF occupancy. Gray saturation is proportional to the maximum signal strength observed in any cell line. Colors in the histone mark tracks (H3K4Me1, H3K4Me3, H3K27Ac) represent conventional ENCODE codes used to illustrate different cell lines.

Supplementary Table S1: Primers used for PCR. A. Primers used for RT-qPCR to quantify mRNA levels. **B.** Primers used for the PCR-mediated amplification and cloning of ERE1 to 4 of the first intron of the *EPAS1* gene

A

mRNA	Primer	Sequence	Length
HIF-2α	forward	5’-TTGATGTGGAAACGGATGAA-3’	196
	reverse	5’-GGAACCTGCTCTTGCTGTTC-3’	
HIF-1α	forward	5’-TCCGATGGAAGCACTAGACA-3’	243
	reverse	5’-TGGTGACAACTGATCGAA-3’	
ERα	forward	5’-TCTTGGACAGGAACCAGGAG-3’	315
	reverse	5’-TGTGGGAGAGGATGAGGA-3’	
PgR	forward	5’-GGTCTACCCGCCCTATCTCA-3’	151
	reverse	5’-GCTCCCACAGGTAAGGACAC-3’	
CITED-2	forward	5’-GGAGCAGAAATCGCAAAAAC-3’	334
	reverse	5’-GACCCATGAACTGGGAGTTG-3’	
L28	forward	5’-GCAATTCCTTCCGCTACAAC-3’	198
	reverse	5’-TGTTCTTGCGGATCATGTGT-3’	

B

ERE	Primer	Sequence	Length
ERE1	forward	5’-TCCCCCGGGTTGTCCTATCCTCGGAGCAC-3’	496
	reverse	5’-CCGCTCGAGTCTTTGGCAAACAACCAAAA-3’	
ERE2	forward	5’-TCCCCCGGGGTGGAAACCAGTTTGGCTGT-3’	201
	reverse	5’-CCGCTCGAGGCCCTCTAAGACTGCCTGTG-3’	
ERE3	forward	5’-TCCCCCGGGTTGATACCTGAATGGCCACA-3’	503
	reverse	5’-CCGCTCGAGTGCCCAGAGCTTCGTTATCT-3’	
ERE4	forward	5’-TCCCCCGGGGGATGTCCAAGCTGCCTTTA-3’	270
	reverse	5’-CCGCTCGAGAGGTCTTCATGCTCCTCCAA-3’	
